# Supplementary material for: Preliminary efficacy of cognitive-behavioral therapy on emotion regulation in adults with autism spectrum disorder: A pilot randomized waitlist-controlled study
Source: PLoS One. 2022 Nov 23;17(11):e0277398. doi: 10.1371/journal.pone.0277398 (PMC9683545; doi:10.1371/journal.pone.0277398)
Supplement: S2 File — (PDF) [file pone.0277398.s002.pdf]

ASD Knowledge and Attitude Quiz

Name: ______________________

I. Please answer the following questions by circling “yes,” “no,” or “DK” (don’t know).

**1**	The cause of developmental disorders is the way that parents raise children.	Yes	No	DK
**2**	The cause of developmental disorders is bullying.	Yes	No	DK
**3**	Autism Spectrum Disorder (ASD) includes Asperger Syndrome.	Yes	No	DK
**4**	Individuals with ASD don’t have characteristics of ADHD.	Yes	No	DK
**5**	Developmental disorders including ASD involve different brain types from typical development.	Yes	No	DK
**6**	Individuals with ASD become depressed when bad things happen. Typically developed individuals do the same.	Yes	No	DK
**7**	No individuals with ASD have sensory problems.	Yes	No	DK
**8**	Individuals with ASD are good at working in the hospitality business.	Yes	No	DK
**9**	Individuals with ASD can do routine work, but they can’t adapt to changes of circumstances flexibly.	Yes	No	DK
**10**	Many individuals with ASD can work free of anxiety and independently, if they have a visual schedule.	Yes	No	DK

Please turn over.

□. Please circle the number that best fits your opinion.

		1 definitely disagree	2 slightly disagree	3 not sure	4 slightly agree	5 definitely agree
**1**	It is not fair that individuals with ASD make concessions to typically developed people all the time.	1	2	3	4	5
**2**	The best life for individuals with ASD is not the same as for typical development, but is adjusted to the character of ASD.	1	2	3	4	5
**3**	The difference between ASD and typical development is not large, but about the same as differences in blood type.	1	2	3	4	5
**4**	The good aspects of typical development are different from ASD. Therefore, their collaboration will create a better world.	1	2	3	4	5
**5**	There have been great inventions and work completed by the autistic characteristics of individuals with ASD.	1	2	3	4	5

Please check for missing responses. Thank you very much.
