# Supplementary material for: Preliminary efficacy of cognitive-behavioral therapy on emotion regulation in adults with autism spectrum disorder: A pilot randomized waitlist-controlled study
Source: PLoS One. 2022 Nov 23;17(11):e0277398. doi: 10.1371/journal.pone.0277398 (PMC9683545; doi:10.1371/journal.pone.0277398)
Supplement: S3 File — (PDF) [file pone.0277398.s003.pdf]

(ver. 2011.02)

STUDY PROPOSALS

(Date created: Feb.4th, 2011)

Title of investigation	A Randomized Controlled Trial of a Cognitive-Behavioral Intervention for Emotion Regulation in Adults with High-functioning Autism Spectrum Disorders
Study Director	Yuki Kawakubo, Ph.D. (assistant professor)<br>Affiliation: Department of Child Neuropsychiatry, Graduate School of Medicine, The University of Tokyo.<br>7-3-1, Hongo, Bunkyo-ku, Tokyo, 113-0033, Japan<br>Tel 03-3815-5411(33622), FAX 03-5800-6894<br>E-mail: yukik-tky@umin.ac.jp
Co-Researchers (Name, Affiliation, Role)	Miho Kuroda : Department of Child Neuropsychiatry, Graduate School of Medicine, The University of Tokyo; Department of Psychology & Shukutoku University (contributes to the design and management of this trial and wrote most of the manuscript)<br>Hitoshi Kuwabara: Department of Child Neuropsychiatry, Graduate School of Medicine, The University of Tokyo (contributes to the design and management of this trial and wrote most of the manuscript)<br>Kazuhito Yokoyama: Department of Epidemilolgy and Environmental Health, Graduate School of Medicine, Juntendo University (contributes to the development of the CISS-Japanese version)<br>Yukiko Kano: Department of Child Neuropsychiatry, Graduate School of Medicine, The University of Tokyo<br>Yoko Kamio: Department of Child and Adolescence Mental Health, National Institute of Mental Health, National Center of Neurology and Psychiatry.<br>YKs are the directors of each site and make substantial contributions to revising the design and management of this trial.
collaborating research institutes	Department of Child and Adolescence Mental Health, National Institute of Mental Health, National Center of Neurology and Psychiatry
Duration	Aug. 1st, 2011~Dec. 31st, 2015

[Background]

Adults with autism spectrum disorders (ASD) often show the “theory of mind” deficit toward other individuals. Recent studies suggest that they cannot understand their own minds either (Attwood, 2004; Szatmari et al., 2008; Williams, 2010) and accordingly cannot exercise self-regulation. Some studies have reported the effectiveness of cognitive-behavioral therapy (CBT) for improving self-regulation, especially that related to anxiety, in children and teenagers with ASD (Sofronoff et al., 2005, 2007; White et al., 2009; Wood et al., 2009). However, there have been few studies in adults with ASD.

[Objectives]

The purpose of this study is to investigate the efficacy of group-based CBT for adults with high-functioning ASD. Our primary hypothesis is that adults with ASD can understand their own emotions, exercise self-regulation, and thus alleviate their own secondary symptoms such as anxiety and depression.

[Method]

Study Design:

This is a randomized controlled trial. Flow diagram of subject progress through the phases of the randomized trial is below. Enrollment and random allocation will be performed through central registration at the University Hospital Clinical Trial Alliance Clinical Research Supporting System (UHCT ACReSS) at the University of Tokyo. A minimization method will be used with sex as the allocation factor. A third party, who is not involved in this trial, will enroll participants after examining their eligibility and informed consent. Owing to allocation concealment, the random allocation sequence will be provided by UHCT ACReSS and will not be revealed to any researchers or staff until the end of the enrollment period. As this is a single-blinded trial, all assessments will be conducted by raters without knowledge of whether the participant is in the CBT or waitlist conditions.

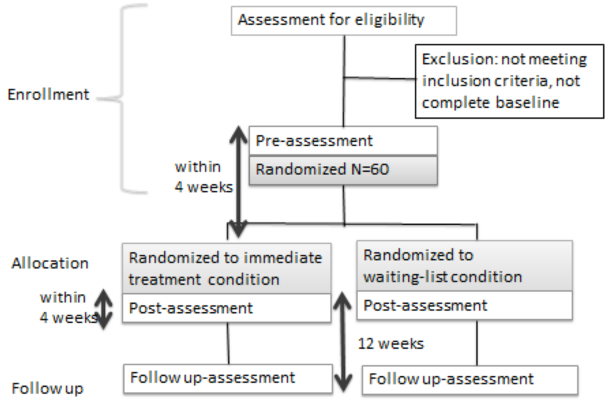

Figure 1: CONSORT flow chart of the clinical trial

Participants

CBT group:&Control group: 30 adults with ASD each group, totally 60 adults

<u>Inclusion Criteria</u>

- Aged 18-50 years
- Primary diagnosis of ASD (based on DSM-IV-TR criteria by psychiatrists) a full IQ of at least 85 and a verbal IQ of around 100 and above
- ADOS or ADI-R score above the ASD cut-off point
- Educational qualifications: high school graduated or higher
- Individuals that were informed their diagnosis
- Aware that he or she has poor emotional self-awareness or ability to express emotion and is poor at understanding others' emotions and thoughts

<u>Exclusion Criteria</u>

- psychiatric comorbidity and unstable condition

Measures

<u>primary outcome measures</u>:

- The TAS-20 (Toronto Alexithymia Scale) : One of the most commonly used measures of alexithymia. This measure is a self-report one and consists of 20 items and 3 factors.
- The MPMR (Motion Picture Mind-Reading task) : This is the advanced theory of mind tasks, which consists of a total of 41 video clips. The participant is asked to judge whe ther the word presented on the screen aptly describes the person in each scene.
- The CISS(Coping Inventory for Stressful Situations) : It determines the preferred copin g style of an individual and to assess the relationship between the individual's coping s tyle and his or her personality.
- The ASD questionnaire : It was developed for this study to assess the knowledge about ASD and the attitude to ASD.

<u>secondary outcome measures:</u>

- The STAI (State-trait Anxiety Inventory): Self-report questionnaire that includes separat e measures for state and trait of anxiety.
- The SPAI (Social Phobia and Anxiety Inventory) : Self-report questionnaire that assess es specific somatic symptoms, cognitions, and behaviors across a wide range of potential ly fear-inducing situations to measure social anxiety and fear.
- The LSAS (Liebowitz Social Anxiety Scale) : Questionnaire designed to assess the range of social interactions and performance situations that individuals with social phobia m ay fear and/or avoid. This measure was designed as a self-report questionnaire, but w e use it here in the form of an interview.
- The CES-D (Center for Epidemiological Studies Depression): Self-report screening tool

of depression.

- The GAF (Global Assessment of Functioning): It is used by clinicians to make a global assessment of an individual's adaptive level of functioning .
- The WHO-QOL 26 (World Health Organization Quality of Life 26-item): It is used to m easure an individual's subjective sense of wellbeing and quality of life, rather than dete rmining the possible presence of an illness.

All assessment will be administrated by the blinded assessors at the enrolment, post-inte rvention, and at a 12 weeks follow-up

Intervention program

CBT group received group therapy over a 8-week period (1 session/week) with each session lasting approximately 100 min. Group therapy consisted of 4~5 adults with ASD and 2 psy chologists. We are using the visual materials for this program, mainly the Cognitive Affecti ve Training Kit (CAT-kit) (Attwood, 2008). Also, we developed the original teaching materia ls for psychoeducation on ASD.

- Attendance rate under 75 % was treated as drop out.

Statistical methods

All analyses will be performed using SPSS 20 J (SPSS Inc., Chicago, IL, USA). All data will be analyzed under the intent-to-treat principle. For the primary outcomes, independen t *t*-tests will be used to compare changes in scores between the pre-assessment and post-as sessment periods between the CBT group and the waitlist control group. The primary outco mes will be analyzed controlling for potential confounds (e.g., age, gender, IQ, and clinical c haracteristics) using regression models. Secondary outcomes will be analyzed using relevan t tests at each assessment, controlling for possible confounds as described above. Subgroup analyses will be performed for any possible confounds to differentiate the efficacy of CBT at follow-up.

[Methods of Publication] : publication in academic journals & conference presentation

[Ethical Considerations]

- Privacy protection(protection of human rights for the participants):

Linkable anonymizing. The information including a name, an address, a telephone number, etc., which can discriminate an individual is deleted, and attaches an original number and which can be connected is conducted. An individual cross reference list is kept in the cabin et which can be locked in a laboratory, and a chief researcher has responsibility. After the end of research, the all data is discarded by a shredder. All data is analyzes in the PC

which is not connected to the internet using the password which only the researchers of this research can know. Since all data is statistically processed, an individual is not specifi ed in papers or presentations.

- Consideration of the safety and disadvantage on participants:

Each time, observation, and consultation are carried out. When any problem is seen in a participant, he/she are introduced to the University of Tokyo Hospital or the primary physician.

- Informed consent

All participants will be asked to sign a written informed consent, as approved by the ethical committee of each site, according to the Declaration of Helsinki after receiving a complete explanation of the trial.

[Research Fund]

This study is supported by a Grant-in-Aid for Scientific Research(C) (No 22531078), the Japan Society for the Promotion of Science (JSPS), Japan; Intramural Research Grant (23-1) for Neurological and Psychiatric Disorders of the National Center of Neurology and Psychiatry (NCNP); and a Grant-in-Aid for Scientific Research on Innovative Areas (No. 23119706), Ministry of Education, Culture, Sports, Science and Technology (MEXT), Japan.
