# Supplementary material for: Preliminary efficacy of cognitive-behavioral therapy on emotion regulation in adults with autism spectrum disorder: A pilot randomized waitlist-controlled study
Source: PLoS One. 2022 Nov 23;17(11):e0277398. doi: 10.1371/journal.pone.0277398 (PMC9683545; doi:10.1371/journal.pone.0277398)
Supplement: S4 File — (PDF) [file pone.0277398.s004.pdf]

様式第1□ 1号

研　究　倫　理　審　査　申　請　書

平成　　　年　　月　　日

東京大学医学系研究科長・医学部長　殿

申請者（研究責任者） a)　氏名　川久保友紀　　　　　　　　　印

所属・職名　医学系研究科こころの発達医学分野・助教

電話　37644　　E-mail　Yukik-tky@umin. ac. jp

下記の研究について、倫理審査を申請いたします。

記

研究課題	発達障害の診断および心理的介入法の有効性に関する検討
キーワード（５つ程度）	ランダム化単盲検介入研究、発達障害、多施設共同研究、質問紙調査、侵襲性無、
研究従事者の<br>氏名・所属・職名等	（氏名）　（所属）　（職名）　（研究倫理セミナー受講No及び年月日）b)<br>川久保友紀　大学院医学系研究科こころの発達医学分野　助教　H23.1.28, H22-25-082<br>山末英典　大学院医学系研究科臨床神経精神医学分野　准教授　H21.9.14, H21-21-347<br>桑原 斉　大学院医学系研究科こころの発達医学分野　助教　H21.9.24, H21-21-206<br>戸所綾子　大学院医学系研究科こころの発達医学分野　大学院生　H21.5.15, H21-20-199<br>石井 礼花　大学院医学系研究科小児医学分野　助教　H22.6.1,　H22-23-455<br>金生 由紀子　大学院医学系研究科こころの発達医学分野　准教授　H22.9.22, H22-24-273<br>原　郁子　こころの発達診療部　特認臨床医　H22.6.1, H22-23-225<br>浅見　綾　こころの発達診療部　心理士　H23.5.24, H23-26-294<br>神尾陽子　国立精神・神経医療研究センター精神保健研究所児童・思春期精神保健部　部長<br>黒田美保　国立精神・神経医療研究センター精神保健研究所児童・思春期精神保健部　研究員<br>Latha V Sooya　Mt.Sinai School of Medicine　Assistant Professor<br>田中康雄　北海道大学大学院教育学 研究院附属子ども発達臨床研究センター　教授<br>[illegible]
連絡担当者	氏名：川久保友紀（かわくぼゆき／Kawakubo Yuki）<br>所属・職名：大学院医学系研究科こころの発達医学分野　助教<br>電話：（内線 33622）（ＰＨＳ37644）・E-mail：yukik-tky@umin.ac.jp
添付書類一覧	資料１：説明文書（1-1 発達障害患者用／1-2 家族用）　資料２：同意書（2-1 発達障害患者用／2-2 家族用）<br>資料３：研究計画書<br>資料４：心理的治療プログラムの内容<br>資料５：倫理委員会承認通知書

a)研究責任者は常勤教職員に限る。大学院生・研究生は研究責任者になることはできない。
b)研究責任者は研究倫理セミナー受講証の写を必ず添付すること（研究責任者は受講していない場合、審査を受けられない。また学内研究従事者も原則として研究開始前までに受講する必要がある)。
尚、外部施設所属の者は除く。

研　究　計　画　書

１．研究課題　発達障害の診断および心理的介入法の有効性に関する検討

２．研究の概要

２・１　背景及び目的

広汎性発達障害や注意欠如／多動性障害などの発達障害は、乳幼児期から学童期に発症し、生涯続く疾患であり、養育者等から得られた幼少期の発達経過に関する情報と現在の様子の行動観察により診断されている。英語圏では、構造化された面接法や観察法、標準化された質問紙などの診断ツールが多数存在するが、国内で使用可能なツールはほとんどない。そのため、養育者の情報が十分でない場合には診断が困難であったり、面接や行動観察を行なう者によって診断が一貫しなかったりするなど、診断の妥当性の問題がある。また、治療においては、本人自身や家族が障害特性を理解し、適切な対処行動を身につけていくための心理的介入が不可欠であるが、その効果や手法についての検証が不十分である。

そこで、本研究では、以下のことを目的とする。１）構造化面接法／観察法や質問法を用いて発達障害を診断し、その妥当性を検証する。２）広汎性発達障害に対し、心理的介入を行ない、構造化面接法／観察法や質問紙法、認知機能検査を用いてその介入効果を検証する。

２・２　方法

広汎性発達障害や注意欠陥多動性障害等の発達障害患者を対象として、自己記入式および他者記入式の質問紙法や本人あるいは養育者への面接法や観察法を用いて診断を行なう。構造化診断面接場面は、実施方法の妥当性を複数名で検討するため、ビデオに記録する。さらに、行動特徴（CBCL, QOL, CES-D, CISS）、性格傾向（STAI, SPAI, LSAS, TAS20）, 認知機能（WISC, WAIS, DN-CAS,WCST,CPT, 心の理論課題）を評価する。場合によって、東京大学医学部倫理委員会に承認されている脳MRI検査（受付番号 397-1、承認済み）、機能的MRI検査（受付番号1350、承認済み）を行い、脳形態や機能との関連を検討することがある。それらの際には、それぞれの検査について改めて説明・同意の手続きを経た上で行う。

広汎性発達障害成人への小集団介入プログラムの介入効果を検証するため、診断後、認知行動療法による心理的介入が行なわれ、介入群と統制群とで症状や行動特徴、性格傾向, 認知機能を群間比較する。その際、候補者をランダムに介入群と統制群に割り振り、評価者に盲検をかけたランダム化比較試験とする。ただし、倫理面に配慮し短期効果測定後に統制群についても介入群と同じ介入プログラムを実施する。活動実施後にビデオ映像を用いて内容の記録をとるため、さらに、医師や心理士等に心理社会的介入プログラムの内容などを教育する際の視覚教材として使用するため、ビデオ録画を行なう。

実施期間	承認後　5　年間	研究期間	承認後　5　年間

２・３　対象及び資料等

１）対象 発達障害（広汎性発達障害(60名)、AD/HD(50名)）を対象とする。基本的には、研究への協力の同意が得られた東京大学医学部附属病院精神神経科に通院中または入院中の患者を対象とするが、公募等で自由意志に基づいた参加希望があれば、医療機関にかかっていない当事者や他院にかかっている患者も対象とする。被験者には、研究の主旨、目的について充分な説明を行って同意を得る。対象とする疾患の発症や受診の年齢を考慮して年齢制限は2歳以上とし、治療効果については18歳以上に絞った対象とする。性別は問わない。研究期間は承認後5年間を予定している。これらの研究参加を呼びかける際には、自由意思による協力を求め、強制力が働かないように十分配慮する。

２）　資料等　アンケートの回答、認知機能検査、ビデオ映像

２・４　研究参加者（被験者・研究対象者）の実体験

心理的治療の内容については、別紙資料を参照。

2歳～17歳の発達障害患者

1）倫理審査委員会で認められた説明文書により、適切かつ十分な説明を受ける。自由意思に基づいて、研究参加への同意を表明した後、質問紙法や面接法により診断を受ける（120分）。別途の同意が得られた場合、面接場面をビデオ録画し、海外の共同研究機関に送付する。

2）行動特徴および性格傾向についての質問紙への記入を行う（30分）。

18歳以上の広汎性発達障害患者（介入あり群）

1）倫理審査委員会で認められた説明文書により、適切かつ十分な説明を受ける。自由意思に基づいて、研究参加への同意を表明した後、質問紙法や面接法により診断を受ける（120分）。別途の同意が得られた場合、面接場面をビデオ録画し、海外の共同研究機関に送付する。

2）行動特徴および性格傾向についての質問紙への記入を行う（30分）。

3）認知機能を評価する心理検査では、コンピューター画面を見てボタンを押したり、カードなどの簡単な用具を用いて指示に応じて行動したりする（30分）。1回では終了しない場合には、約1ヶ月以内に残りの検査などを受ける。

4）脳MRI検査（受付番号 397-1、3048承認済み）、機能的MRI検査（受付番号629-1、3048承認済み）を各倫理規程に従い、受けることがある

5）3～8名程度の患者に対し2～4名程度のスタッフで構成される心理社会的介入を週1回1時間半程度、2ヶ月間受ける。介入内容の記録のため、ビデオ録画する。

6）介入期が終了した時点、さらにその3ヶ月に、再度１）～3）を受ける。場合によっては、4）も受ける。

7）別途、同意が得られた場合、介入場面のビデオ映像を医師や心理士等の教育に用いる。

18歳以上の広汎性発達障害患者（心理的治療なし群）

1）倫理審査委員会で認められた説明文書により、適切かつ十分な説明を受ける。自由意思に基づいて、研究参加への同意を

表明した後、質問紙法や面接法により診断を受ける（120分）。別途の同意が得られた場合、面接場面をビデオ録画し、共同研究機関に送付する。

2） 行動特徴および性格傾向についての質問紙への記入を行う（30分）。

3） 認知機能を評価する心理検査では、コンピューター画面を見てボタンを押したり、カードなどの簡単な用具を用いて指示に応じて行動したりする（30分）。1回では終了しない場合には、約1ヶ月以内に残りの検査などを受けることがある。

4） 脳MRI検査（受付番号 397-1、承認済み）、機能的MRI検査（受付番号629-1、承認済み）を各倫理規程に従い、受けることがある

5） 2ヶ月後、6ヶ月後、再度1）〜3）を受ける。場合によっては、4）も受ける。

３．研究を実施する施設とその役割

１）該当する本学および学外施設名とその役割（別途添付可）

①インフォームド・コンセントを受ける施設：被検者の都合や状態に応じて、東京大学医学部附属病院南研究棟、精神神経科外来、B 病棟１階こころの発達診療部、昭和大学医学部附属烏山病院

②個人情報及び資料等を収集又は所有する施設：東京大学医学部附属病院南研究棟、精神神経科外来、B 病棟１階こころの発達診療部、昭和大学医学部附属烏山病院、国立精神・神経医療研究センター

③資料等を匿名化する施設：東京大学医学部附属病院南研究棟１階第１研究室

④資料等を解析する施設：東京大学医学部附属病院南研究棟１階第１研究室、国立精神・神経医療研究センター、北海道大学大学院教育学 研究院附属子ども発達臨床研究センター、浜松医科大学、順天堂大学、福島大学

⑤資料等を保存する施設：東京大学医学部附属病院南研究棟１階第１研究室、国立精神・神経医療研究センター、北海道大学大学院教育学 研究院附属子ども発達臨床研究センター、浜松医科大学、順天堂大学、福島大学

２）（該当する場合）学外施設での対応とその状況（別途添付可）

すでに国立精神・神経医療研究センター（受付番号、19-4-事7）、北海道大学大学院教育学 研究院附属子ども発達臨床研究センター（受付番号08-24）、昭和大学医学部附属烏山病院、浜松医科大学（第22-25号）、順天堂大学（受付番号693）、福島大学（受付番号23–10）の倫理委員会にて承認されている。

４．研究における倫理的配慮

４・１　インフォームド・コンセント

１） 実施方法

研究参加者に対し、Helsinki宣言に則り、別紙説明書を用いて、面談形式で、研究の趣旨、内容、および本研究に同意しない場合でも不利益を受けないこと、同意の撤回が可能であることなどを説明し、別紙同意書に沿って承諾を得る。その際、強制力が働かないよう十分配慮する。

特に倫理的な配慮を必要とする研究参加者への配慮の有無と対応策　→　■あり（内容を記入）　□なし

ありの場合は、該当項目の番号を○で囲み、対処する方法を記入すること。

１．未成年者　２．成人で十分な判断能力のない場合　３．成人で意識のない場合　４．その他　例えば病名に対する配慮が必要な場合

未成年者や、成年でも精神神経疾患のために十分な判断能力がないと考えられる場合には、保護義務者に対して説明を行い同意が得られれば検査を行う。その場合にも、可能な限り本人からも同意を得る。

４・２　個人情報保護

１）本学における個人情報の有無とその種類　→　■あり　□なし

資料等・同意書等における氏名・生年月日・顔の画像・音声データが個人情報に該当する。

２）個人情報保護の方法

検査データ及び解析結果は姓名を連結可能匿名化して、東京大学医学部附属病院南研究棟１階第１研究室内の鍵のかかる保管庫で保管し、電子データは LAN につながれていないパスワードロックのかかるスタンドアローンのパソコンに保管する（データの管理責任者：川久保友紀）。構造化診断面接の実施方法について、共同研究者からスーパーバイズを受けるため、ビデオを共同研究機関（マウントシナイ大学、国立精神・神経医療研究センター）へ送付する。映像データの送付については、研究参加とは別に同意を得た上で、名前などの個人情報を削除し、ファイルに暗号化処理を施して送付される。介入／面接場面のビデオ映像は、研究への参加とは別に同意を得た上で、医師や心理士等の教育のために用いられる。

３）研究期間終了後：個人情報の保存／廃棄方法

東京大学医学部附属病院南研究棟１階第１研究室内の鍵のかかる保管庫で保管し、電子データは LAN につながれていないパスワードロックのかかるスタンドアローンのパソコンに保管する（データの管理責任者：川久保友紀）。

４・３　個人情報を含まない資料等の取扱

東京大学医学部附属病院南研究棟1階第１研究室内に保存する。当該研究課題の範囲外で使用したり、当該研究従事者以外が使用したりする場合には改めて東京大学倫理委員会の承認を受けた上で用いることとする。

５．安全の確保

１）研究によって研究参加者に生じうる危険や不快等

①診断面接、質問紙への記入、認知機能検査が長時間に及ぶことによる、疲労や不快、②検査や面接を個別で受けることに対する不安感、③心理社会的介入プログラム（別紙、資料４）への参加や対人交渉に対する抵抗感が生じる可能性がある。

２）危険や不快等への対応策

①長時間に渡る検査により被験者に疲労が見られた場合は、途中に適宜、休憩をはさむ。その上で、検査の続行を被験者が拒否した場合には検査をいったん中止し、2回に分けて行なう。

②本人あるいは保護者が希望すれば、検査に保護者の立会いを認め、不安などの軽減に努める。

③心理的介入時に被験者に異常が認められた場合、即座に精神科医に連絡をとる体制を整える。心理的介入を被験者が拒否した場合には心理的介入を中止する。

３）研究参加者に対する研究結果の開示

個人情報が明らかにならないように配慮した上で、学術集会や学術誌上で結果を公表することがある。臨床症状の評価結果は診療上で有用と思われるものは、診療録に報告を保存し、診療担当医の判断で、各個人の臨床症状の評価結果を告知することがある。

６．　備　考

研究参加者に対しては、検査にかかる時間に応じて、時給1,000円として謝礼を算出し、交通費（1,000円）を合わせて支払う。ただし、2ヶ月間の心理的介入参加に対しては、謝礼と交通費を含め1回の参加につき1,000〜1,500円支払う。必要な経費は、文部科学省科学研究補助金、科学研究補助金厚生労働省、戦略的創造研究推進事業、寄付金によってまかなわれる。

診療科長または教室責任者　　氏名

（自署に限る。捺印省略可）

病院長　　　　氏名　　　　　　　　印

（附属病院でおこなわれる研究の場合）
