# Supplementary material for: Preliminary efficacy of cognitive-behavioral therapy on emotion regulation in adults with autism spectrum disorder: A pilot randomized waitlist-controlled study
Source: PLoS One. 2022 Nov 23;17(11):e0277398. doi: 10.1371/journal.pone.0277398 (PMC9683545; doi:10.1371/journal.pone.0277398)
Supplement: Attachment — Submitted filename: Response_to_Reviewers.docx [file pone.0277398.s007.docx]

We would like to thank the reviewers for their insightful comments and suggestions, which have helped us improve our manuscript and provide a more balanced account of our research. We have carefully reviewed our manuscript in light of their comments and made the necessary changes, which are shown in red font in the revised manuscript to facilitate the review process. Please find below our point-by-point responses to their comments.

Reviewer #1

*Line 335, Repeated measures ANOVA comes with pairwise/multiple comparison for post hoc test. If based on repeated measures ANOVA approach, the use of t test to be clarified in the text.*

**Response:** Thank you for your valuable suggestion. We have revised our manuscript accordingly (page 21, line 305; page 24, line 333).

Reviewer #2

*I still feel the discussion and abstract needs to be more forthcoming describing the general lack of change across the majority of measures. Overall, I think that, although more work and research is needed, the discussion needs to be cautious in suggesting that there may only minimal value in group CBT for autistic adults based on findings in this study.*

**Response:** Thank you for your insightful comment. We have revised the discussion section (page 28, lines 405-407), and added the following explanation of the general lack of differences between groups to both the discussion section and the abstract of our revised manuscript to be more forthcoming:

“However, the modest and inconsistent effects underscore the importance of continued efforts to improve the CBT program program beyond current standards” (page 4, lines 23-24; pages 28-29, lines 421-423).

*An additional outstanding concern is the use of language. When I refer to identity first, I mean using the term “autistic adult” not “ASD adult” or “ASD individual”. Alternatively neutral language, which you could perhaps use at the start of the introduction, would be “Adults on the autism spectrum”. Also I think it’s better to refer to autism generally after an initial first mention of ASD.*

**Response:** Thank you for this valuable comment. In response to your comment, we changed the description to “autistic” instead of “ASD” throughout our revised manuscript.

*Just checking if it should be an ‘or’ not an ‘and’ in the list of tools used in diagnostic confirmation?*

**Response:** We apologize for the mistake. It should be “or”. We have revised the manuscript accordingly (page 9, line 126).

*In the analyses section you say no adjustment was made for multiple comparisons, then in results a Bonferroni correction is described?*

**Response:** We appreciate your comment. We deleted the following sentence from the analyses section: “We did not conduct a correction for the multiple comparisons due to the exploratory nature of this study.”

*Significance is unclear to me when it is reported “With 356 respect to the TAS20-F2 scores, a significant effect of treatment group was found (F = 5.81, p = 0.19)” given the p value?*

**Response:** We apologize for the mistake. It should be “*p* = 0.019”. We have revised the manuscript accordingly (page 24, line 347).
