# Supplementary material for: Preliminary efficacy of cognitive-behavioral therapy on emotion regulation in adults with autism spectrum disorder: A pilot randomized waitlist-controlled study
Source: PLoS One. 2022 Nov 23;17(11):e0277398. doi: 10.1371/journal.pone.0277398 (PMC9683545; doi:10.1371/journal.pone.0277398)
Supplement: Attachment — Submitted filename: Response_to_Reviewers.docx [file pone.0277398.s008.docx]

We would like to thank the reviewers for their insightful comments and suggestions, which have helped us improve our manuscript and provide a more balanced account of our research. We have carefully reviewed our manuscript in light of their comments and made the necessary changes, which are shown in red font in the revised manuscript to facilitate the review process. Please find below our point-by-point responses to their comments.

Reviewer #2

*Thank you for addressing previous suggestions. I noticed - "it remains unclear which aspects of the CBT program are associated with changes in ASD" - I don't believe ASD changes and feel this sentence should be revised.*

**Response:** We apologize for the mistake. It should be “emotion regulation”. We have revised the manuscript accordingly (page 28, line 413).
